# Searches as data: archiving and sharing search strategies using an institutional data repository

**DOI:** 10.5195/jmla.2024.1791

**Published:** 2024-01-16

**Authors:** Alisa B. Rod, Jill T. Boruff

**Affiliations:** 1 alisa.rod@mcgill.ca, Research Data Management Specialist, Research and Scholarship, McGill University Library, McGill University, Montreal, QC, Canada; 2 jill.boruff@mcgill.ca, Co-Lead Editor, Journal of the Medical Library Association, Associate Librarian, Schulich Library of Physical Sciences, Life Sciences, and Engineering, McGill University, Montreal, QC, Canada

**Keywords:** Research Data Management, Data Deposit, Data Repository, Knowledge Synthesis, Expert Searching, Research Reproducibility, Systematic Review Methodology

## Abstract

**Background:**

By defining search strategies and related database exports as code/scripts and data, librarians and information professionals can expand the mandate of research data management (RDM) infrastructure to include this work. This new initiative aimed to create a space in McGill University's institutional data repository for our librarians to deposit and share their search strategies for knowledge syntheses (KS).

**Case Presentation:**

The authors, a health sciences librarian and an RDM specialist, created a repository collection of librarian-authored knowledge synthesis (KS) searches in McGill University's Borealis Dataverse collection. We developed and hosted a half-day “Dataverse-a-thon” where we worked with a team of health sciences librarians to develop a standardized KS data management plan (DMP), search reporting documentation, Dataverse software training, and howto guidance for the repository.

**Conclusion:**

In addition to better documentation and tracking of KS searches at our institution, the KS Dataverse collection enables sharing of searches among colleagues with discoverable metadata fields for searching within deposited searches. While the initial creation of the DMP and documentation took about six hours, the subsequent deposit of search strategies into the institutional data repository requires minimal effort (e.g., 5-10 minutes on average per deposit). The Dataverse collection also empowers librarians to retain intellectual ownership over search strategies as valuable stand-alone research outputs and raise the visibility of their labor. Overall, institutional data repositories provide specific benefits in facilitating compliance both with PRISMA-S guidance and with RDM best practices.

## BACKGROUND

Prior to the development of public data repository infrastructure, researchers commonly relied on publishers to archive any data underlying their publications [1]. Despite the development of data repositories, researchers continue to share data in the attached appendices or supplemental materials of related journal articles [2, 3]. When data are shared as supplemental materials it is typically the publishers who retain full intellectual ownership (i.e., copyright) [4]. Alternatively, researchers may have opted to indicate within their publication(s) that they would share the data upon request. However, generally, researchers subsequently fail to facilitate data transfers or ensure data are preserved long-term for this purpose. The inaccessibility of research data contributed to the reproducibility crisis in many fields [5–7], including knowledge synthesis [8].

Over the past 10 years, researchers have been facing increasing pressures and incentives to openly share, via distinct preservation-oriented repository platforms, any data that underly published research findings, articles, or other scholarly works [9, 10]. Indeed, major public funders (e.g., the US National Institutes of Health (NIH), the National Science Foundation, the Canadian Tri-Agency, etc.) are requiring, or phasing in requirements, for research data underlying publicly funded studies to be FAIR (i.e., that data underlying research publications should be findable, accessible, interoperable, and reusable) [11–13]. The NIH's 2020 Data Management and Sharing Policy, effective since January 2023, requires grant recipients to “plan and budget for the managing and sharing of data” [12]. The Canadian Tri-Agency harmonized Research Data Management Policy is phasing in a requirement for grant recipients “to deposit into a digital repository all digital research data, metadata and code that directly support the research conclusions in journal publications and pre-prints that arise from agency-supported research” [13].

In addition, journal publishers are requiring data availability statements or commitments from authors that data underlying publications will be deposited [3]. The International Committee of Medical Journal Editors (ICMJE) requires that researchers submitting manuscripts to ICMJE journals must provide a data sharing statement indicating whether the data will be available, where the data will be deposited, and which components or versions of the data will be shared [14]. A recent study by Ngyuen et al. concludes that “journal policies on data sharing might encourage sharing of review materials” [15].

In this way, the research community increasingly view data as important research outputs separate from any related publications [16]. In addition, the FAIR principles for research data are accepted as the best practice across many scholarly disciplines, funding bodies, and journals/publishers [11]. Although the FAIR principles are not completely synonymous with the open science or open data movements, they are highly related in terms of prioritizing the reproducibility of research findings based on empirical evidence as the cornerstone of research transparency and integrity.

In general, the current best practice among research data management (RDM) professionals is to define research data as all the information that is required to reproduce the findings of a study or to verify the findings of a study [17–19]. In this way, a dataset may include computational scripts or code, a codebook or data dictionary, metadata, and other related documentation. For example, in order to replicate the findings of a study, it may be necessary to understand the process by which data were transformed from their original raw state into a clean version, who collected the data, what is the source of the data, who owns the data, and whether there are any limitations regarding the data collection. Thus, a dataset may be composed of many interrelated components including all iterations of the dataset as well as the final version.

With this conceptualization of datasets, a search strategy created for a knowledge synthesis (KS) project can be seen as the code used to retrieve data (the list of relevant abstracts or citations) from a database. This framework suggests that search strategies and related output files are functionally equivalent to research datasets. Librarians who collaborate on KS projects by developing search strategies and exporting records from abstracting and indexing databases are creating intellectual work that contains inherent value as a research output separate from any related publications [20–22].

In addition, according to PRISMA-S guidance, “authors should upload complete documentation to a data repository, an institutional repository, or other secure and permanent online archive instead of relying on journal publication” [23]. Thus, expanding the mandate of RDM infrastructure to include search strategies allows librarians and information professionals who work on KS projects to take advantage of the features of these infrastructure systems to make KS searches compliant with reporting procedures (e.g., PRISMA-S) and professional best practices [24]. This case study presents a new initiative of health sciences librarians and the RDM specialist at McGill University to identify the appropriate repository, create documentation, and populate the repository in order to comply with PRISMA-S guidance for archiving search strategies and curate, preserve, and raise visibility of a collection of librarian-authored KS work [25].

## CASE PRESENTATION

McGill University in Montréal, Québec, Canada is a large, publicly funded research institution with a team of seven health sciences librarians collaborating on KS projects across all health sciences disciplines. The team needed a way to better document and share their searches among colleagues and for publication. The health sciences librarian author approached the RDM specialist author to discuss the possibility of using the institutional data repository for these purposes. Before settling on the institutional repository, the authors discussed the needs of the librarians for their KS deposits and examined all feasible repository options. We wanted to ensure that our choice followed the FAIR principles, including increased discoverability, accessibility, interoperability, and reusability, through features such as the minting of persistent identifiers (e.g., DOIs) for each unique object, indexing across major search engines or databases, allowing for the deposit of preservation file types (i.e., open formats instead of proprietary file formats), and allowing for the assignment of an appropriate digital and legally binding license (e.g., a Creative Commons license).

We examined several existing platforms that may be used to archive or publish searches to weigh their benefits and disadvantages. Open Science Framework (OSF) is commonly used for preserving data, documents, and KS work [26]. Launched in late 2021, there is also a domain-specific pre-print style repository, SearchRxiv, that incorporates curation into the workflows for publishing search strategies [27]. Finally, there are institutional repositories that are oriented towards building collections of research outputs by affiliates of a given institution. For example, the University of Michigan is using their institutional data repository, Deep Blue Data, to store and preserve KS work produced by their institutionally affiliated librarians [28].

Following a review of relevant repositories and archives, we decided to focus on institutional options for this initiative for several reasons. First, one key goal of our initiative was to create and maintain a collection produced by McGill University librarians in order to demonstrate the impact of our individual and collective KS work. Oftentimes, the intellectual work of the librarians on these KS projects was unrecognized, particularly when KS projects stalled and never got published.

In our perspective, work by a KS researcher at one institution is likely to be more relevant to other KS researchers at the same institution. Also, if all McGill University librarian search strategies are organized and preserved in one place, it is easier to share our work with each other, demonstrate the quantity of work produced by our group of librarians, and easily collect evidence of reuse. Second, our librarians aimed to maintain at least some degree of curatorial control over their deposited search strategies. Finally, our institutional platforms are free to use, are built on open-source software, and have stable long-term funding and contingency plans.

We decided not to use our institutional repository (IR), as it is designed primarily for completed documents, including theses, post-prints, and other types of manuscripts. In this way, the IR is not equipped with the robust metadata needed for archiving search strategy documentation as well as the ability to have several versions of the same record (for when a search is changed upon update, for example). In addition, our IR does not have the capability to issue DOIs, nor does it accept a wide variety of file types, but rather is restricted to document file types (e.g., .pdf, .docx, etc.).

We instead chose to use the institutional data repository, which uses the Dataverse software, an open-source repository platform originally developed by the Institute for Quantitative Social Science (IQSS) at Harvard University [29]. Since the Borealis Dataverse installation represents a Canada-wide shared infrastructure service, each institutional Dataverse collection (e.g., McGill University Dataverse) are nested hierarchically under a top-level Borealis Dataverse collection [30]. The Dataverse software allows for each repository collection to contain sub-collections which all may contain one or more datasets. Datasets may contain files, documentation, and metadata.

Borealis Dataverse issues DOIs, incorporates extensive discoverable metadata fields, and allows for the deposit of all types of files and documentation. In addition, the Borealis Dataverse platform is fully bilingual and can be operated in both English and French, which is an important feature for Canadian institutions. Finally, the institutional Dataverse allows for restricted access or access control on individual files. In this way, health sciences librarians can deposit export files from proprietary databases and mediate access to institutional affiliates, thus avoiding a violation of vendor terms while maintaining replicability and reusability.

Once we chose the platform, we created a sub-collection in the McGill collection of Borealis named “McGill Librarian Knowledge Synthesis Search Repository,” to make clear that it contains only the work of librarians. Our deposits are strictly the searches, and the metadata links to the resulting publication (when relevant).

The authors then organized a three-hour “Dataverse-athon” with the intention of co-creating guidance and documentation to standardize KS deposits and to begin depositing KS search strategies. In January 2022, the authors led a three-hour session, which was held virtually due to COVID-19 restrictions in place at that time. During the “Dataverse-a-thon,” the McGill University health sciences librarians worked with the RDM specialist to develop a draft data management plan (DMP) and README document outlining standardized file formats, file naming conventions, licenses/copyright issues, and a template for inputting metadata fields [31, 32]. DMPs are “living documents” and are updated as new situations for their application arise. Given the novelty of our approach, we anticipate regular updates to our documentation. The most recent version of the DMP/README file and the search reporting template can be found at https://doi.org/10.5683/SP3/FNRHJ2. In the same session, we conducted a training on how to upload and publish data to the institutional Dataverse, co-created sub-collections within the institutional Dataverse for each liaison area (e.g., psychiatry, rehabilitation, dentistry, nursing, etc.), and provided administrative permissions to each corresponding liaison librarian over their own sub-collection. Co-creating the documentation, including a standardized README document and DMP, took almost the entire duration of the initial three-hour “Dataverse-athon” session. However, this process allowed all the participating librarians to talk through how they would be using the Dataverse and ensured that the file naming conventions and metadata entries for the data deposits were applicable to different types of KS work.

To build on the momentum from the initial session, we held a second 2-hour session in the spring of 2023 that was focused primarily on helping librarians deposit and publish KS searches. At that time, we also decided to combine the DMP and README files into one document, so it could serve as a quick reference when librarians are depositing datasets. Currently, there are 6 published subject sub-collections within the McGill University librarian KS collection and 23 published datasets. Overall, there were 233 file downloads across these 23 datasets as of July 2023, and the dataset containing our DMP/README documentation has been downloaded 114 times. The full McGill University librarian KS collection can be found at https://borealisdata.ca/dataverse/mcgill_librarian_ks_search_repository.

## DISCUSSION

While the initial creation of the DMP and README took some time, the subsequent deposit of search strategies into the institutional data repository has been low effort and quite successful. We are now able to deposit the complete documentation from each KS project, with a typical deposit consisting of a PRISMA-S compliant document with all complete search strategies from all databases in one file, the RIS files or other database output files, and other documentation related to a project. All data are stored according to industry standards for cybersecurity (e.g., encrypted at rest), on servers located at Canadian academic institutions in Ontario, and users aiming to deposit data must authenticate through institutional affiliations [33]. Metadata are searchable across our KS sub-collection and harvestable by search engines and other repositories [33]. This functionality facilitates discoverability and provides a low barrier for finding our own work in the future, either for our own reuse or to share with colleagues.

Depositing search strategies in an institutional data repository takes 5-10 minutes, on average, per deposit, once a workflow is established, while also potentially reducing the mental load of how to name, store, and find existing searches. For institutions or librarians interested in launching a similar initiative, we recommend launching the projects with two 2-hour sessions, with one session dedicated to training and documentation co-creation, and one session where librarians should be prepared to deposit at least one KS search strategy. Our practice of depositing our searches has been accepted by our research teams, especially when we explain that KS searches translate their research questions into a script that retrieves data according to a set of parameters that the researchers require in making claims for evidence-based studies. We recognize, however, that not all researchers will necessarily be so quick to accept this practice, and it is a question we hope to explore in future research.

In general, institutional data repositories, or institutional Borealis Dataverse collections in the Canadian context, provide specific benefits in facilitating compliance both with PRISMA-S guidance and with RDM best practices. Librarians can deposit the complete search strategy document in RTF format (the preservation standard format), as well as all the direct database downloads, for full transparent reporting of the data retrieval. Database downloads are often RIS files, which are proprietary, and they may be deposited in Borealis Dataverse collections using a feature that allows for restricting file access (i.e., access control). The Dataverse software also allows for versioning of records, meaning that librarians can update the search strategy over time and maintain a record of changes. In addition, the Dataverse software allows for the application of a license or terms of use. In practice, this means that the author(s) of a search strategy can determine to what extent, and in what contexts, their search(es) can be reused for other research projects. If the author(s) of a search strategy license(s) their work openly, Dataverse generates a citation that makes it easy for others to cite their work when it is reused in other projects, which can provide evidence of the broader impact of their intellectual work. Finally, there should be no need to duplicate efforts if a search strategy already exists that can answer or contribute toward answering a new research question, which parallels the notion that there should not be a need to collect the same dataset multiple times.

Since presenting this project at the Canadian Health Libraries Association's 2021 annual conference, three other institutions in Canada have launched their own librarian KS sub-collections in their institutional Borealis Dataverse collections modeled on our work [25, 34–36]. We are collaborating with these librarians to survey health sciences librarians across Canada on sharing their KS data to inform our work with other institutions across Canada to build standardized collections of deposited searches.

This initiative illuminates the distinct benefits of using an institutional data repository to archive KS search strategies. Librarians can retain intellectual ownership over search strategies as stand-alone research outputs and prevent errors that often can be introduced during the journal publication process. The search strategies will no longer be buried in supplemental files or behind journal paywalls. We hope that deposits in a data repository will help to answer Ross-White's question “What does it mean when we replace the vocabulary of librarianship (search) with the more male-dominated language of computer science (algorithm)?” [20]. By considering our searching as coding, and depositing it as such, we endeavor to make the invisible visible, since librarian work continues to be poorly documented in published reviews [20, 37]. While a KS manuscript may reduce the librarian's work to just a few sentences, the search strategy document in a deposit allows a librarian to fully describe and record every decision made in their search. A librarian can then deliver their documentation to the research team through a link to their work, and this link makes it easier for the research team to include the complete search documentation in a manuscript. Even if a librarian is not given authorship or an acknowledgement by the authors, the link will lead anyone who looks at the search strategy to the librarian. While this is not a perfect solution, it can be a step in making librarian labor more visible. These dataset citations can also be used to demonstrate the amount of labor and impact a librarian has to supervisors and administrators and is not dependant on the publication by a research team. In this way, an institutional data collection of librarian-authored search strategies provides a comprehensive resource, via a single URL, to illustrate the breadth of librarian contributions. Overall, based on our experience, maintaining a librarian collection of search strategies as datasets may increase the broader visibility of the distinct value added by librarian contributions to KS projects.

## Data Availability

Data associated with this article are available in the McGill University Dataverse at https://doi.org/10.5683/SP3/T7ITRM.
